# The Impact of Amlodipine on Gingival Enlargement After Kidney Transplantation

**DOI:** 10.5812/numonthly.5427

**Published:** 2012-06-20

**Authors:** Zohreh Rostami, Behzad Einollahi, Mohammad Javad Einollahi, Simin lessan

**Affiliations:** 1 Nephrology and Urology Research Center, Baqiyatallah University of Medical Sciences, Tehran, IR Iran; 2 Oral Medicine Department, Islamic Azad University-DentalBranch, Tehran, IR Iran

**Keywords:** Amlodipine, Gingival Overgrowth, Kidney Transplantation

## Abstract

**Abstract:**

Background: Although cyclosporine (CsA) and calcium channel blockers (CCBs) parallel to each other may provoke gingival enlargement (GE), there are few considerations about combined effects of CsA and CCBs on gingival tissues.

Objectives: This study aimed to determine prevalence of GE among renal transplant recipients and to compare its occurrence in patients who received only CsA and those who were on CsA and amlodipine.

Patients and Methods: We conducted a prospective randomized case-control trial including 213 renal transplant recipients between February 2010 and August 2010. They were randomly divided into two groups including control group (on continuous treatment with CsA alone; n = 112) and trial group (treated with combined CsA and amlodipine; n = 101). Buccal, lingual, and inter-proximal membranes at last 12 anterior teeth were assessed for GE and packet depth (PD) using Gingival Index of McGaw and others, and Packet Index of Turesky–Gilmore–Glickman, respectively.

Results: Marked GE was observed in 26 patients (25.7%) in trial group and only in 4 individuals (3.6%) in control group (P = 0.000). In logistic regression analysis, obese (OR = 3, P = 0.04), older (OR = 2.8, P = 0.03), and female (OR = 1.3, P = 0.03) recipients as well as who received high dose amlodipine (OR = 4.4, P = 0.000) were at risk for marked GE.

Conclusions: There is a strong correlation between GE, in particular marked GE, and combination therapy with CsA and amlodipine in transplant patients compared to those treated by CsA alone. We suggest CsA dose reduction may restrain this adverse effect.

## 1. Background

Cyclosporine (CsA) has been widely prescribed to prevent graft rejection after kidney transplantation (1). However, it may cause side effects that may clinically become prominent such as gingival enlargement (GE) and blood hypertension (1-3). Hypertensive episodes that frequently occur in this setting are often treated by calcium channel blockers (CCB) such as amlodipine (4). Moreover, amlodipine significantly increases calcineurin inhibitor level in hypertensive renal transplant recipients (5).

Drug induced GE is a clinical condition characterized by an increase in the size of gingival tissues leading to an alteration in gingival contour (6). It could be the side effect of several drugs such as anticonvulsants, immunosuppressive agents, and calcium channel blockers (7). Gingival enlargement is considered as the most common periodontal side effect of amlodipine (6). On the other hand, incidence of GE in renal transplant recipients maintained on CsA varies from 13 to 84.6% (1, 8, 9).

## 2. Objectives

Although CsA and CCB parallel to each other may provoke GE, there are few considerations about combined effects of CsA and CCB on gingival tissues. Therefore, this study aimed to determine prevalence of GE among renal transplant recipients and to compare its occurrence in patients who received only CsA and those who were managed by CsA and amlodipine.

## 3. Patients and Methods

### 3.1. Participants

We conducted a prospective randomized case-control trial including 213 renal transplant recipients to evaluate correlation between amlodipine and GE. Living and deceased kidney transplants recipients were both included. Eligible subjects had at least six maxillary and six mandibular anterior teeth with stable renal function and maintained on triple immunosuppressive therapy (including CsA, mycophenolate mofetil, and prednisone). Subjects were excluded if they exhibited poor oral hygiene, had a history of kidney transplantation less than 3 months, managed by anticonvulsant drugs or oral contraceptives, suffered from major illnesses, or were pregnant. They were randomly divided into two groups of control (on continuous treatment with CsA alone; n = 112) and trial (on combined treatment with CsA and amlodipine; n = 101). The current study protocol was approved by the local Ethics Committee of Baqiyatallah University of Medical Sciences and an informed consent was obtained from all patients.

### 3.2. Demographic and Biochemical Data Collection

The clinical data collected for all patients included age at transplantation and at diagnosis, gender, body mass index (weight/hieght2), CsA dose, GE, PD, and amlodipine dose and treatment duration . Biochemical data such as plasma creatinine concentration, and C0 (through) and C2 (2 hour post dose) blood levels of CsA were also assessed.

### 3.3. Definition

Marked PD and marked GE were defined as PD more than 1.5 and GE more than 2.5.

### 3.4. Immunosuppressive Regimen and Follow up

Immunosuppression was maintained in all patients based on CsA plus mycophenolate mofetil, and prednisolone. The amount of cyclosporine given to transplant patients was mostly tailored by blood levels of drug (10, 11). CsA monitoring using its blood levels was periodically performed at different times and the dose was adjusted if necessary. Cyclosporine was targeted at 150-250 ng/mL through blood level during 3 months and tapered subsequently to 100-150 ng/mL by 1 year, while we performed C2 target levels 800 to 1000 ng/mL in month one to three after transplantation, and C2 target levels 400 to 600 ng/mL thereafter. Control patients were only maintained on CsA microemulsion but trial patients while continued to receive CsA, amlodipine was added to control their hypertension at initial dose of 2.5 mg twice daily with subsequent dose adjustment.

During February 2010 to August 2010, all patients were examined for periodontal status by an expert periodontist who was blinded to both control and trial groups. Buccal, lingual, and inter-proximal membranes at last 12 anterior teeth were assessed for GE and packet depth (PD) using Gingival Index of McGaw and others (12), and Packet Index of Turesky–Gilmore–Glickman (13), respectively.

### 3.5. Statistical Analysis

Statistical analysis was performed using SPSS version 17.0 for Windows. All quantitative variables have been expressed as mean ± SD and the qualitative variables have been shown by percentage. Continuous data were compared by Student’s t-test and Mann-Withney, and categorical data were analyzed by Chi-square or Fisher’s exact test. Univariate and multivariate analysis were performed to evaluate correlation between GE and PD with confounding factors using a logistic model. Independent variables with P values ≤ 0.2 in univariate analysis were entered into a multivariate logistic regression model. P values less than 0.05 were considered statistically significant.

## 4. Results

### 4.1. Patient Demographics

No significant differences were observed between two groups in terms of gender, age at transplantation, age at diagnosis of GE, BMI, and renal allograft function (Table 1). The mean age of all transplant patients was 34.6 ± 12.8 years (range: 6-69 years) at the time of transplantation, and 39.8 ± 12.6 years (range: 9-71years) at diagnosis time of GE. The mean plasma creatinine concentration was 1.4 ± 0.5 mg/dL (range: 0.64-3.3 mg/dL). The average elapse time between transplantation and gum changes was 64.0 ± 53.8 months (range: 12-276 months). Eligible subjects were treated by CsA on average dose of 155 ± 40 mg/day (range: 50-275 mg/day). Average dose of amlodipine in trial group was 6.8 ± 2.4 mg/day (range: 5-10 mg/day) for 24 ± 12 months (range: 3-74 months).

**Table 1:** Comparison Between Control and Trial Groups

	**Trial **	**Control **	**P value**
Age at transplantation, mean±SD, y	35.04 ± 11.7	39.1 ± 13.7	0.6
Age at diagnosis, mean±SD, y	39.8 ± 11.3	39.9 ± 13.8	0.9
Gender, No. (%)			0.2
Male	54 (53.5)	68 (60.7)	
Female	47 (46.6)	44 (39.3)	
Creatinine, mean±SD, mg/dL	1.4 ± 0.4	1.5 ± 0.5	0.2
C0^b^ level, mean±SD, ng/mL	103 ± 40	119 ± 56	0.02
C2^c^ level, mean±SD, ng/mL	439 ± 109	502 ± 144	0.001
CsA dose, mean±SD, mg/d	154 ± 37	156 ± 43	0.7
BMI ^a^, mean±SD	26.1 ± 5.18	25.5 ± 5.3	0.4
GE ^a^, mean±SD	0.9 ± 0.75	0.5 ± 0.55	0.000
Marked GE, mean±SD, No. (%)	26 (86.7)	4 (13.3)	0.000
PD ^a^, mean±SD	2.6 ± 0.9	2.7 ± 0.9	0.5
Marked PD, No. (%)	61 (45.5)	73 (54.5)	0.4

^a^Abbreviations: BMI, body mass index; GE, gingival enlargement; PD, packet depth

^b^C0, blood trough level of CsA

^c^C2, 2 hours post-dose blood level of CsA

### 4.2. Comparison Between the Groups

As a whole, no significant differences were observed between two groups unless about GE which was higher in trial group (0.9 ± 0.75 vs. 0.5 ± 0.55, P = 0.000), and C0 and C2 levels which were more significant in control group (P = 0.02 and P = 0.001); however, the doses of CsA in both groups were not significantly different (Table 1). Frequency of of GE and PD did not show significant differece in male and female patients (Table 2).

**Table 2:** GE and PD in Different Gender

	**Male**	**Female**	**P value**
GE^a^, mean ± SD	0.7 ± 0.62	0.7 ± 0.67	0.9
PD^a^, mean ± SD	2.7 ± 0.93	2.7 ± 94	0.5

^a^Abbreviations: GE, gingival enlargement; PD, packet depth

The correlation between marked GE and marked PD with CsA blood levels in trial and control groups were summarized in Tables 3 and 4. Although C0 and C2 levels were lower in patients with marked GE as compared to those who had no marked GE, this difference was only significant for C0 blood level in trial group (P = 0.02); moreover, CsA blood levels in control patients were greater than in trial individuals with no significant differences (Table 3). No correlation was observed between CsA blood levels and marked PD among both groups (Table 4).

**Table 3:** Marked GE Comparison Between Trial and Cotrol Groups with Consideration C0 and C2 Levels

	**Number **	**Mean **	**P value**
Trial			
C0^b^ level			0.02
No Marked GE^a^	75	108 ± 42	
Marked GE	26	90 ± 30	
C2^c^ level			0.1
No Marked GE	75	449 ± 117	
Marked GE	26	411 ± 73	
Control			
C0 level			0.2
No Marked GE	107	118 ± 57	
Marked GE	4	134 ± 23	
C2 level			0.2
No Marked GE	99	499 ± 146	
Marked GE	3	569 ± 68	

^a^Abbreviation: GE, gingival enlargement

^b^C0, blood trough level of CsA

^c^C2, 2 hours post-dose blood level of CsA

**Table 4:** Comparison Marked PD Between Trial and Cotrol Groups with consideration C0 and C2 Levels

**Group**	**CsA Level**	**Severity of PD ^a^**	**No.**	**Mean ± SD**	**P value**
Trial					
	C0 ^b^ level				0.9
		No Marked PD	40	103 ± 43	
		Marked PD	61	104 ± 38	
	C2 ^b^ level				0.8
		No Marked PD	40	437 ± 118	
		Marked PD	61	441 ± 103	
Control					
	C0 level				0.1
		No Marked PD	39	108 ± 59	
		Marked PD	72	125 ± 54	
	C2 level				0.08
		No Marked PD	37	469 ± 139	
		Marked PD	65	520 ± 145	

^a^Abbreviations: PD, packet depth

^b^C0, blood trough level of CsA; C2, blood concentration of CsA at two hours after administration

### 4.3. Marked GE and Marked PD

The mean amount of GE and PD were 0.72 ± 0.6 (0-2.7) and 2.7 ± 0.9 (0.33-4.8), respectively; whereas marked GE and marked PD were observed in 14% (n = 30) and in 63% (n = 134) of all patients, respectively. In addition, marked GE was seen in 26 (25.7%) patients of trial group but in only 4 (3.6%) individuals in control group (P = 0.000). Furthermore, marked PD was observed in 61 (60.4%) cases in trial group and in 73 (65.2%) subjects in control group (P = 0.4). We also found a marked PD in older recipients (36.2 ± 13 vs. 31.8 ± 12, P = 0.01). Higher doses of amlodipine was significantly associated with marked GE (8.4 ± 2.3 vs. 6.2 ± 2.2, P = 0.000).

### 4.4. Univariate Analysis

At univariate analysis in trial group, PD enhanced with increasing dose of amlodipine (0.008); also GE was correlated with high dose of amlodipine (P = 0.000) and greater BMI (P = 0.01) (Table 5). Furthermore, a dose response curve between gingival enlargement and amlodipine (curve 1) was observed.

**Table 5:** Univariate Correlation Between PD a; and GE a and Contributing Factors in Trial Group

	**PD, r (P value)**	**GE, r (P value)**
Age at transplantation	0.1 (0.08)	0.1 (0.1)
Age at diagnosis	0.1 (0.09)	0.1 (0.1)
CsA dose	0.01 (0.1)	0.007 (0.9)
Time duration of amlodipine	0.1 (0.1)	0.1 (0.1)
Amlodipine dose	0.2 (0.008)	0.4 (0.000)
Creatinine level	0.08 (0.4)	0.02 (0.8)
BMI ^a^	0.1 (0.1)	0.2 (0.01)

^a^Abbreviatins: BMI, body mass index; GE, gingival enlargement; PD, packet depth

Although marked GE was significantly correlated with higher doses of amlodipine (P = 0.000), this correlation was modest in lower levels of CsA (Table 6). Higher rate of marked PD occurred in kidney transplant recipients with advanced age (Table 7).

**Table 6:** Participated Variables in Logistic Regression Model for GE a

	**Marked GE**	**Mild GE**	**Univariate(P value)**	**MultivariateOR (P value)**
Age at transplantation, mean±SD, y	38.2 ± 14	34.02 ± 12	0.1	2.8 (0.03)
Transplantation until diagnosis, mean±SD, y	4.06 ± 2.3	5.4 ± 4.8	0.1	-
BMI, mean±SD	27.1 ± 6.1	25.5 ± 5	0.1	3 (0.04)
C0^b^level, mean±SD, ng/mL	96 ± 33	114 ± 52	0.06	2.4 (0.006)
C2^c^ level, mean±SD, ng/mL	427 ± 86	477 ± 136	0.05	-
Amlodipine dose, mean±SD, mg/d	8.4 ± 2.3	6.2 ± 2.2	0.000	4.4 (0.000)
Gender, No. (%)			0.09	1.3 (0.03)
Male	13 (43.3)	109 (59.6)		
Female	17 (56.7)	74 (40.4)		

^a^Abbreviations: BMI, body mass index; GE, gingival enlargement

^b^C0, blood trough level of CsA

^c^C2, blood concentration of CsA at two hours after administration

**Table 7:** Participated Variables in Logistic Regression Model in Marked PD

	**Marked PD**	**Mild PD**	**Univariate (P value)**	**Multivariate OR (P value)**
Age at transplantation, mean±SD, y	36.7 ± 13.0	31.8 ± 12.1	0.01	2.8 (0.01)
Age at diagnosis, mean±SD, y	41.2 ± 13.0	37.5 ± 11.8	0.03	-
BMI^a^, mean±SD	26.2 ± 5.5	25.1 ± 4.6	0.1	-
C0^b^ level, mean±SD, ng/mL	115 ± 48	105 ± 51	0.1	2.4 (0.02)
C2^c^ level, mean±SD, ng/mL	481 ± 132	452 ± 129	0.1	-
Amlodipine dose, mean±SD, mg/d	7.1 ± 2.4	6.3 ± 2.3	0.08	4 (0.000)
Cyclosporine dose, mean±SD, mg/d	158 ± 38	149 ± 43	0.1	-

^a^Abbreviation: BMI, body mass index

^b^C0, blood trough level of CsA

^c^C2, blood concentration of CsA at two hours after administration

### 4.5. Multivariate Logistic Regression

Obese (OR = 3, P = 0.04), older (OR = 2.8, P = 0.03), and female (OR = 1.3, P = 0.03) recipients as well as who received high doses of amlodipine (OR = 4.4, P = 0.000) were at risk for marked GE (Table 6).

The older age (OR = 2.8, P = 0.01), high C0 level (OR = 2.4, P = 0.02), and high dose of amlodipine (OR = 4, P = 0.000) were risk factors for marked PD (Table 7).

## 5. Discussion

Drug induced GE is a well-documented complication in renal transplant recipients. The highest prevalence of GE is reported among those treated with CsA and CCBs (76%) (3), consistent with its prevalence in our renal recipients. The main objective of this study was to evaluate the effect of amlodipine on GE and periodontal breakdown. Major finding of the present study was the strong correlation between GE, in particular marked GE, and combination therapy with CsA and amlodipine in transplant patients when compared to those treated with CsA alone. Drug induced GE prevalence varies among studies. General prevalence rates of CsA- associated GE vary from 8% to 81% (14).

Advance reports have revealed that more severe gingival changes developed in patients who received a combination of CsA and CCB than those who treated only by CsA (3, 15, 16). The prevalence rate of GE occurred when amlodipine was used alone has been shown to be between 1.7% and 3.3% (2, 17, 18).

Drug induced GE should be treated based on the medication being used and clinical presentation of each individual. First of all, we have to consider the possibility of ceasing or replacing the drug. Simple withdrawal of offending agent is usually not a practical decision. However, its replacement with another medication might be a practical solution (1). CsA discontinuation is the simplest approach to reduce GE (2).

In our transplant center, we monitor recipients with CsA blood levels and its side effects. In the presence of CsA side effects, dose reduction is the major approach. According to Rodwan et al. study in 2003 (19) CsA dosage was a risk factor for GE (4). However, we found marked GE in lower C0 and C2 levels likewise higher dose of amlodipine. It emphasizes that the effect of amlodipine in hypertrophy induction was more prominent than that of CsA and that it was independent to CsA. By the way, in this condition CsA dose reduction was one of the most logical approaches. Shiboski et al. (2009) (20), Reali et al. (2009) (21) and Lima et al. (2008) (22) reported that male gender was more susceptible to GE than female. However, we found no correlation between GE prevalence and its severity with gender of recipient, time since transplantation, CsA dose, and last creatinine level, except in higher BMI values. Similar to Torrezan et al. (2005) (23), GE induced by CsA was more frequent in obese recipients; in addition, in this study likewise Thomas et al in 2001 study (24) GE was unrelated to allograft function.

However, the prevalence of GE in our patients was lower than that in report of James et al. (16) showing a prevalence of 72% with CsA and amlodipine treatment (2, 25). It might be due to different size of sample or different related prescribed doses. On the other hand, we emphasize that although amlodipine shows great GE prevalence when used in combination with CsA, this combination displays lower GE prevalence and severity than that revealed in CsA-nifedipine combination (2, 6). In addition, other risk factors were not significantly effective on periodontal lesions in the current study.

The fact that marked PD was more dominant in all recipients and in control group than marked GE, motivate a theory that possibly PD – compared to GE - was more influenced by CsA This theory might be supported by the evidence that in trial group which –compared to control group - CsA was prescribed in lower dose, occurrence of PD was less dominant. In contrary, lower prevalence of GE in all recipients and control group, may support this theory that amlodipine potentially induce dose dependent GE more frequent than PD. It seems that the extent of PD depends on other contributing factors, too.

### 5.1. Limitation

There are so many potential risk factors such as genetic susceptibility, oral hygiene, and demographic, pharmacologic, and periodontal variables which contribute GE and PD (26-28). In Rodwan et al. study (19) it was shown that HLA-DR2 phenotype was a risk factor for GE; nevertheless, we did not include genetic factors in our survey. Therefore, a large prospective randomized control trial is still required.

We suggest co-management of hypertension by other drugs such as aldosterone receptor blockers or CCBs with lower side effects like diltiazem that did not induce GE (29). Prescribing CsA along with a CCB should be limited. Although the majority of studies offer CsA replacement with tacrolimus (30-32), if it is not possible, we suggest CsA dose reduction that may restrain this adverse effect.
